# Dental Students’ Satisfaction With Web-Based Learning During the Initial Phase of the COVID-19 Pandemic: Mixed Methods Study

**DOI:** 10.2196/50278

**Published:** 2024-03-08

**Authors:** Minjung Lee, So Youn An, Jungjoon Ihm

**Affiliations:** 1 Yale School of Nursing Yale University New Haven, CT United States; 2 College of Dentistry Wonkwang University Iksan Republic of Korea; 3 Dental Research Institute, School of Dentistry Seoul National University Seoul Republic of Korea

**Keywords:** web-based learning, student satisfaction, transactional distance theory, health profession education, COVID-19

## Abstract

**Background:**

The COVID-19 pandemic has precipitated an accelerated shift in education, moving from traditional learning to web-based learning. This transition introduced a notable transactional distance (TD) between the instructors and learners. Although disease control and staff and students’ safety are the top priorities during a pandemic, the successful delivery of education is equally crucial. However, the ramifications of this swift transition are particularly critical in the context of dental education. Dental education is inherently practice oriented, necessitating hands-on training and manual skills development, which poses unique challenges to distance learning approaches.

**Objective:**

This study aims to examine dental students’ web-based learning satisfaction and experience of TD, investigate the predictors of web-based learning satisfaction, and explore the perceptions of students about the advantages and disadvantages of web-based learning.

**Methods:**

This study explored the factors associated with web-based learning satisfaction using TD theory during the transition to web-based education. Psychological factors that could influence satisfaction were adapted from the health belief model. We conducted a cross-sectional web-based survey of 345 dental students from 2 institutions in South Korea who were enrolled in the spring semester of 2020. Data were collected between July 8 and September 14, 2020. Qualitative analysis was used to examine responses to open-ended questions concerning perceptions of web-based learning.

**Results:**

A multivariate hierarchical linear regression model was used to analyze the effects of student characteristics, TD, and psychological factors (ie, perceived risk of infection and efficacy belief of web-based learning) on web-based learning satisfaction. The average score for web-based learning satisfaction was 3.62 (SD 0.84), just above the midpoint of the possible range (1-5). Self-regulated learning (β=0.08; *P*=.046), learner-instructor interaction (β=0.08; *P*=.03), and learner-content interaction (β=0.64; *P*<.001) were associated with higher levels of satisfaction. Moreover, a significant association was revealed between high efficacy beliefs in web-based learning (β=0.20; *P*<.001) and satisfaction. Although the learning structure (synchronous vs asynchronous) did not exhibit a significant association with satisfaction, the qualitative analysis results revealed that each structure possesses distinct strengths and weaknesses. The students in synchronous learning (79/345, 22.9%) recognized heightened autonomy in the “learning environment” (19/79, 24%); however, technical issues (28/79, 35%) and reduced concentration (15/79, 19%) were identified as downsides. Conversely, the students in asynchronous settings (266/345, 77.1%) emphasized unlimited access to learning content (74/266, 27.8%) and the flexibility of “learning in preferred time” (69/266, 25.9%). Nevertheless, challenges, such as self-management difficulties (66/266, 24.8%) and limited interactions (55/266, 20.7%), were evident.

**Conclusions:**

The findings suggest that efforts to minimize TD, facilitating self-regulated learning and interaction among students and instructors, are critical for achieving web-based learning satisfaction. Moreover, establishing a common understanding among students regarding the necessity and efficacy of web-based learning during epidemics could enhance their satisfaction.

## Introduction

### Background

The battle to combat COVID-19 has resulted in an unprecedented public health crisis globally. At the early stage of the pandemic, with heightened concerns owing to the scarcity of clinical measures and the emergence of precautionary behaviors, social distancing and surveillance were considered essential for public safety [1]. Education was no exception; educational institutions were forced to close to prevent virus transmission by eliminating or limiting the contact between students and instructors. These changes transformed classroom education into web-based learning via recorded or live lectures and web-based platforms. Moreover, this change has affected >900 million students globally, including undergraduates in preclinical, clinical, and postgraduate medical education [2-4]. In South Korea, the commencement of the new semester in March 2020 was delayed because of the necessary preparations to accommodate an unforeseen shift to web-based education.

Of particular concern is the field of dentistry, which, given its focus on oral practice, faced unique challenges during the COVID-19 pandemic. The nature of the virus, which is primarily transmitted through respiratory means, exacerbated the situation. Consequently, numerous dental practical classes had to be temporarily postponed to prioritize the safety of both student dentists and patients [5-7]. This adjustment in educational practices has led to increased vigilance within health care professional education in South Korea, with a heightened focus on infection control measures in clinical settings. The necessity of these adaptations underscores the resilience and adaptability of the academic community in responding to the challenges posed by the global health crisis.

The transition from traditional learning to web-based learning driven by the COVID-19 pandemic has affected learning in various fields [8-11]. However, the impact of this transition can be especially crucial for dental education. Dental education is a practice-oriented course of study, which cannot depend on distance learning but requires manual skills training and continuous contact with patients [12-14]. Studies on dental students’ experiences with and perceptions of web-based learning during the COVID-19 pandemic have reported that dental students found web-based learning favorable owing to the effectiveness, safety, and additional time to connect with family [5,12,15]. In contrast, dissatisfaction because of less interaction with other students and instructors [16]; lower quality teaching compared with traditional face-to-face teaching [17]; and high levels of anxiety, depression, and stress [18] were also revealed. Nonetheless, the studies examining the outcomes of web-based learning because of the pandemic and its predictors among dental students remain limited.

The sudden transition from traditional learning to web-based learning driven by the COVID-19 pandemic increased transactional distance (TD). The TD theory defines TD as “the psychological or communicative space that separates the instructor from the learner in the transaction between them, occurring in the structured or planned learning situation” [19]. According to this theory, 3 sets of variables define the pedagogical aspects of education: structure, learner autonomy, and dialogue. Structure is defined as constructs to be learned and measures an “educational program’s responsiveness to learners’ individual needs” [20]. Autonomy is a learner characteristic that refers to self-management during interactions with teachers within a designed structure [21]. Finally, dialogue denotes the interaction between instructors and learners during the implementation of a structured program [22]. This interaction is further classified into learner-content, learner-instructor, and learner-learner interactions. In this study, we adopted Moore’s theory as a theoretical framework to examine TD in a web-based learning environment and its relationship with learning outcomes.

Furthermore, to deepen our understanding and contribution to studies on web-based learning, we adopted psychological factors from health behavior models, such as the perceived risk of COVID-19 and efficacy belief in web-based learning, to prevent the spread of the virus. The health belief model [23] and the extended parallel process model [24] are among the most famous health behavior models, which propose that perceived risk and efficacy beliefs are key contributors in determining individuals’ willingness to make behavioral changes. Multiple studies have reported the robust role of these psychological factors in behavioral responses to reduce the health risk induced by the pandemic [1,25,26]. Although previous studies have revealed a favorable attitude toward the safety and effectiveness of web-based learning [5], perceived risk and efficacy beliefs can relate to the acceptance of the transition from traditional learning to web-based learning and its outcomes.

### Objectives

Disease control and ensuring the safety of students and faculty are the top priorities during any public health crisis. However, the quality and outcomes of education should not be compromised. Furthermore, web-based learning has received substantial attention in the field of dentistry and medicine, and its implementation can offer long-term advantages for dental education beyond the challenges posed by the pandemic. Hence, this study focused on identifying the factors influencing learning outcomes in web-based education, specifically focusing on learning satisfaction among dental students. The objectives of this study are (1) to examine dental students’ web-based learning satisfaction and experience with TD, (2) to investigate the predictors of web-based learning satisfaction, and (3) to explore the perceptions of students regarding the advantages and disadvantages of web-based learning. This study presents several implications for the development and implementation of web-based learning. We believe that the results of this study will provide evidence and implications for guiding the direction of web-based education in the field of medicine and dentistry in the post–COVID-19 era. Furthermore, it will contribute to the development of proactive measures in anticipation of potential public health crises in the future.

## Methods

### Study Design

A cross-sectional web-based survey was designed and conducted during the early stages of the COVID-19 pandemic, inviting students enrolled in the spring 2020 semester to participate. The sample size was calculated using G*Power (latest version 3.1.9.7; Heinrich-Heine-Universität Düsseldorf), which indicated that 315 participants were required to detect small effect sizes (Cohen f^2^=0.08) in linear multiple regression models with up to 10 predictor variables, given a 5% type I error rate and 95% power to calculate the sample size [27].

When this study was conducted, the South Korean government requested that the public minimize face-to-face interactions and isolate themselves at home. Most classes were conducted on the web; potential respondents were invited electronically via email and web-based school bulletin boards. An invitation to participate in the study, including a brief introduction to the background and objective of the study, voluntary nature of participation, and declarations of confidentiality and anonymity, was sent via email to dental students of 2 participating dental schools (N=400). The students who enrolled in the spring 2020 semester at Seoul National University and Wonkwang University in South Korea were invited to participate in this study. These 2 schools differ in several aspects: one is a national institution, whereas the other is a private institution; and one is located in the capital city of Seoul, whereas the other is located in a regional area. In addition, they varied in their approach to web-based classes, with one school using a synchronous format and the other using an asynchronous format. These differences make these 2 schools well suited for gathering a more heterogeneous study sample.

### Data Collection

A survey questionnaire was developed to (1) evaluate dental students’ TD, perceptions, and satisfaction with web-based learning and (2) explore students’ attitudes toward web-based learning driven by the COVID-19 pandemic. An anonymous web-based questionnaire was used to evaluate dental students’ satisfaction with web-based learning. Specifically, it investigated the effects of dialogue on web-based self-regulated learning, perceived risk of COVID-19, and efficacy beliefs about safety and support during web-based learning. Furthermore, the survey included an open-ended question exploring students’ perceived advantages and disadvantages of web-based learning. The survey was conducted using Google Forms. Data were collected from July 8 to September 14, 2020. A total of 345 responses were collected, corresponding to an 86.3% response rate.

### Measurements

#### Satisfaction With Web-Based Learning

Satisfaction with web-based learning was measured using the standardized web-based Online Course Satisfaction Scale (OCSS) [28], which was modified to fit the context of the study. The OCSS presented 7 items on the general contentment level of the students with learning experiences related to instructors and course design. Examples include “I am satisfied with the instructional style” and “I am satisfied with the use of the online discussion forum.” An item on overall satisfaction was included (ie, “Overall, I am satisfied with this course”). Items were rated on a 5-point Likert-type scale ranging from 1 (strongly disagree) to 5 (strongly agree). The Cronbach α value was 0.91.

#### Student Interaction (Learner-Learner, Learner-Instructor, and Learner-Content)

The 3 subscales of interaction (ie, learner-learner, learner-instructor, and learner-content) were adapted from an instrument formulated by Kuo et al [29] to examine student interaction and satisfaction in a blended learning environment. The items were revised to fit the web-based environment of this study. Learner-learner interaction consisted of 8 items that measured the level of communication with fellow students (eg, “I shared my thoughts or ideas about the lectures and their application with other students during this class”). It also included 1 item on overall evaluation (ie, “Overall, I had numerous interactions related to the course content with fellow students”). Learner-instructor interaction consisted of 6 items that measured the level of communication between the learner and instructor and 1 item on overall evaluation (ie, “I asked the instructor my questions through different electronic means, such as email, discussion board, and instant messaging tools”). Finally, learner-content interaction consisted of 4 items (eg, “Online course materials helped me to better understand the class content”). All items in the subscales were measured using a 5-point Likert-type scale, ranging from 1 (strongly disagree) to 5 (strongly agree). The Cronbach α values were 0.92, 0.79, and 0.89, respectively.

#### Metacognitive Self-Regulation

The metacognitive self-regulation subscale of the Motivated Strategies for Learning Questionnaire assessed self-regulated learning and the extent to which students use planning, monitoring, and regulating strategies during learning [30]. A few examples include “When I become confused about something I’m reading for this class, I go back and try to figure it out,” and “I ask myself questions to ensure I understand the material I have been studying in this class.” Items were rated on a 7-point Likert-type scale ranging from 1 (strongly disagree) to 7 (strongly agree). The Cronbach α value was 0.87.

#### Perceived Risk and Efficacy Belief

Regarding psychological factors, we examined the perceived risk of COVID-19 infection (2 items) comprising the perceived susceptibility (individual beliefs about the possibility of infection during face-to-face learning) and the perceived severity of infection [31]. The items were “What do you think is the possibility that you will contract COVID-19 during face-to-face learning?” and “How severe will the COVID-19 infection be?” Items were rated on a 5-point Likert-type scale (1=very low, 3=neither low nor high, and 5=very high). The Cronbach α value was 0.66. The items such as “To what extent do you think online learning is an effective means for reducing the risk of COVID-19 infection?” measured the efficacy beliefs. The items were rated on a 5-point Likert-type scale ranging from 1 (not at all) to 5 (extremely).

Furthermore, we investigated whether the students received the necessary support for web-based learning using 1 of the following resources: lecturer, teaching assistant, classmates, web-based support, or none. To conduct the regression analyses, the responses were converted into binary answers (none=0 and otherwise=1). In addition, the survey included 2 open-ended questions: “What do you think are the advantages of learning online?” and “What do you think are the disadvantages of learning online?”

### Ethical Considerations

This study was approved by the institutional review board of the School of Dentistry, Wonkwang University, Republic of Korea (number WKIRB-202104-SB-021). All methods were performed in accordance with the relevant guidelines and regulations. All participants were informed of their involvement in this study and gave their written consent through Google Forms, agreeing to the anonymous use of their data for publication. The data were collected and analyzed anonymously. No compensation was provided to the study participants.

### Statistical Analyses

Statistical analyses were performed using R software (version 3.5.1; R Foundation for Statistical Computing). The results of the quantitative variables were reported as mean, SD, or frequency (%). A multivariate hierarchical linear regression analysis examined the effects of sociodemographic factors, academic factors, structure, dialogue, self-regulated learning, psychological factors, and web-based learning support on web-based learning satisfaction.

From a qualitative perspective, we conducted content analysis of the responses to the open-ended item. The students’ responses from the 2 dental schools were analyzed separately, given the differences in the web-based lecture structure (synchronous vs asynchronous). All responses to the open-ended item were analyzed inductively with open codes and, subsequently, focus coded using the grounded theory approach [32].

From a qualitative perspective, thematic analysis was used for the written responses on the advantages and disadvantages of web-based learning because of the pandemic [33]. This process aimed to find the main patterns of meaning that occurred across the data and a course of data familiarization, generating codes for features of the data, organizing related codes into main themes, and iteratively reviewing and refining themes to fit the data. The data were analyzed using NVivo software (version 12; QSR International). The first author ML developed a coding frame during the manual coding of responses and identified themes that arose from these codes. For credibility, The authors JI and SYA assessed 50 randomly selected responses and identified their corresponding themes [34]. We reviewed the differences in thematic associations in this subsample and the categorization of codes into themes to reach a consensus.

## Results

### Descriptive Analyses

We collected data from 345 students (male students: 213/345, 61.7%; female students: 132/345, 38.3%), with a mean age of 24.08 (SD 2.68) years (Table 1). The response rate was 86%. In South Korea, dental education operates through 2 parallel tracks: a dental college system comprising a 3-year predental course followed by a 4-year Doctor of Dental Surgery (DDS) degree program and a professional graduate-entry school system that offers a 4-year DDS degree program. The students pursuing the professional graduate-entry system are required to hold a bachelor’s degree before admission, whereas those pursuing the dental college system typically enter with a high school diploma. The students from both systems integrate their studies to achieve the qualification of a dentist with a DDS degree [35,36]. Among the participating schools, 1 school operated simultaneously as a dental college and a professional graduate-entry school system, and the other school operated only as a dental college with a predental program. Among the participants, 84.6% (292/345) of them were from *dental colleges* and 15.4% (53/345) of them were from *professional graduate-entry schools*. The average academic duration was 4.18 (SD 1.54) years. Of the 345 students, 266 (77.1%) and 79 (22.9%) took asynchronous and synchronous web-based classes, respectively.

**Table 1:** Descriptive statistics of the respondents (N=345).

Characteristics			Values
**Sex, n (%)**			
	Male	213 (61.7)	
	Female	132 (38.3)	
Age (years), mean (SD)			24.08 (2.68)
**Academic track, n (%)**			
	Dental college	292 (84.6)	
	Professional graduate-entry school	53 (15.4)	
**Academic period (years)**			
	Value, mean (SD)	4.18 (1.54)	
	≤3, n (%)	107 (31)	
	>3, n (%)	238 (69)	

Table 2 summarizes each scale’s average score and reliability information based on the study sample. The mean OCSS score of the entire sample was 3.62 (SD 0.84), which is close to the midpoint (3) of the possible range (1-5). Each subscale for dialogue had an average score close to the midpoint of the corresponding scale, except for learner-content interaction (mean 3.55, SD 0.86), which exhibited a mean higher than the midpoint. The Cronbach α values were high for each subscale (learner-learner: Cronbach α=0.92 and learner-instructor: Cronbach α=0.79, and learner-content: Cronbach α=0.89). For self-regulated learning, the average score was 4.37 (SD 0.89), with a high Cronbach α value of 0.91. The average score for the perceived risk of COVID-19 infection as a psychological factor was 3.46 (SD 0.99).

**Table 2:** Descriptive statistics of structure, dialogue, self-regulated learning, and psychological factors related to web-based learning during the COVID-19 pandemic (N=345).

Variables		Values
**Structure, n (%)**		
	Synchronous	79 (22.9)
	Asynchronous	266 (77.1)
**Dialogue (5-point Likert scale), mean (SD)**		
	Learner-learner interaction	3.00 (0.86)
	Learner-instructor interaction	3.00 (0.52)
	Learner-content Interaction	3.55 (0.86)
Self-regulated learning (7-point Likert scale), mean (SD)		4.37 (0.89)
**Psychological factors (5-point Likert scale), mean (SD)**		
	Perceived risk of COVID-19 infection	3.46 (0.99)
	Efficacy beliefs of web-based learning on safety	3.99 (1.08)
Web-based learning satisfaction (5-point scale) mean (SD)		3.62 (0.84)
**Web-based** **learning support, n (%)**		
	Instructor and teaching assistant	57 (16.5)
	Classmates	223 (64.6)
	Web-based support	42 (12.2)
	No support	23 (6.6)

The perceived risk of contracting COVID-19 during face-to-face learning was measured using a 5-point Likert-type scale. The average perceived susceptibility was higher than “neutral” (score=3; mean 3.04, SD 1.20). The results indicated that 11.3% (39/345) and 26.4% (91/345) of the students reported the perceived chance of infection as “very high” (score=5) and “high” (score=4), respectively. Many students (105/345, 30.4%) stated that their chance of infection was “half and half.” The average perceived severity score was higher than the perceived susceptibility score, close to “high” (score=4; mean 3.88, SD 1.12). However, 40% (138/345) and 33% (114/345) of the students reported perceived severity as “very high” (score=5) and “high” (score=4), respectively. Alternatively, the average score for efficacy belief about safety was close to “high” (score=4; mean 3.99, SD 1.08). The majority (138/345, 40%) reported that web-based learning effectively reduced the risk of COVID-19 (“extremely”; score=5).

Furthermore, we investigated whether the students received support during web-based learning. The majority (223/345, 64.6%) reported that they received help from classmates, followed by instructors and teaching assistants (57/345, 16.5%) and web-based support through various platforms, such as email, discussion boards, and instant SMS text messaging tools (42/345, 12.2%). However, of the 345 students, 23 (6.6%) reported receiving no support.

### Factors Influencing Students’ Web-Based Learning Satisfaction

Hierarchical linear regression models tested the association between the factors influencing web-based learning satisfaction (ie, web-based course structure, dialogue, self-regulated learning, and psychological factors) and web-based learning support (Table 3). Students’ sex, self-regulated learning, and learner-content interaction (model 1) accounted for 60.6% of the variance. Psychological factors such as the perceived risk of COVID-19 and the efficacy belief of web-based learning in preventing COVID-19 (model 2) explained an additional 3.5% of the variance. Conversely, web-based learning support, as the predictor variable (model 3), explained an additional 1% of the variance in web-based learning satisfaction. In summary, the effect of learner-content interaction (β=0.64; *P*<.001) was significant and the highest, followed by efficacy beliefs of the safety of web-based learning (β=0.20; *P*<.001) and support during web-based learning (β=0.10; *P*<.001). Furthermore, the positive effects of self-regulated learning (β=0.08; *P*=.046) and learner-instructor interaction (β=0.08; *P*=.03) were statistically significant. The effect of the structure of web-based learning was not significant (*P*=.52).

**Table 3:** Multivariate hierarchical linear regression analysis of factors of satisfaction with web-based learning.

Variables^a^	Model 1^b^				Model 2^c^				Model 3^d^		
	Unstandardized coefficient, B	Standardized coefficient, β	*P* value	Unstandardized coefficient, B		Standardized coefficient, β	*P* value	Unstandardized coefficient, B		Standardized coefficient, β	*P* value
Sex (male: 1 and female: 2)	–0.141	–0.082	.02	–0.121		–0.070	.03	–0.118		–0.069	.03
Academic period	0.038	0.069	.06	0.036		0.066	.053	0.031		0.056	.10
Academic track (DC^e^: 1 and professional: 2)	–0.128	–0.055	.32	–0.133		–0.057	.28	–0.154		–0.066	.20
Structure (synchronous: 1 and asynchronous: 2)	–0.062	–0.031	.58	–0.058		–0.029	.59	–0.068		–0.034	.52
Self-regulated learning	0.078	0.083	.047	0.078		0.083	.04	0.076		0.080	.046
Learner-learner interaction	0.010	0.011	.78	0.023		0.023	.53	0.009		0.009	.80
Learner-instructor interaction	0.091	0.078	.06	0.097		0.084	.04	0.098		0.084	.03
Learner-content interaction	0.689	0.703	<.001	0.618		0.630	<.001	0.622		0.635	<.001
Perceived risk of COVID-19	N/A^f^	N/A	N/A	–0.038		–0.045	.18	–0.040		–0.047	.16
Efficacy beliefs of web-based learning on safety	N/A	N/A	N/A	0.161		0.207	<.001	0.154		0.197	<.001
Web-based learning support (none: 0 and otherwise: 1)	N/A	N/A	N/A	N/A		N/A	N/A	0.346		0.103	.001

^a^Nominal variable consists of two options (eg, male and female), and the numerals represent the values assigned to each option.

^b^Model 1: *R*^2^=0.64; Δ*R*^2^=0.606; *P*≤.001.

^c^Model 2: *R*^2^=0.67; Δ*R*^2^=0.035; *P*≤.001.

^d^Model 3: *R*^2^=0.68; Δ*R*^2^=0.010; *P*=.001.

^e^DC: dental college.

^f^N/A: not applicable.

### Perceived Advantages and Disadvantages of Web-Based Learning

Analysis of responses to the 2 open-ended questions, “What do you think are the advantages of learning online?” and “What do you think are the disadvantages of learning online?” exhibited several themes that frequently recurred (Tables 4 and 5).

**Table 4:** Perceived advantages and disadvantages of synchronous web-based learning (n=79).

Theme and perceived advantages and disadvantages			Values, n (%)		Description	Excerpts from written responses
**Advantages**						
	Efficient use of time	27 (34)		Reduction in travel time and time saving		“I like it because I don’t have to spend time traveling to take classes.” “I like being able to use the time before and after class efficiently.”
	The autonomy to choose one’s preferred learning environment	19 (24)		Attend classes from a preferred place (eg, home, coffee shop, or anywhere there is Wi-Fi)		“Taking classes in my own comfortable environment, optimized to learn the most, is the best thing of online learning.” “I can take classes regardless of location.”
	Protective measure during the pandemic	14 (18)		Reduced risk of COVID-19, maintaining social distancing, not a face-to-face learning, and no need to wear facial masks		“It’s a way to escape from the risk of coronavirus infection.” “I feel safe because I don’t have to go to a crowded environment.” “I think it is absolutely necessary to prevent infection in the pandemic era.”
	Heightened concentration	7 (9)		Easier to concentrate and lecture structure is more focused		“As I take classes alone, I seem to be able to concentrate and listen more easily.” “Classes are often held with lecture materials on display, allowing you to concentrate more on the lecture materials while listening to the lecture.”
**Disadvantages**						
	Technical challenges and disruptions	28 (35)		Usability problems (eg, internet cut off during class and electronic devices needed)		“There are times when the instructor’s microphone, speaker, etc. do not work properly.” “If the internet was cut off, lectures would be interrupted, and attendance checks were often not carried out properly.” “If you happen to miss a class due to an internet connection, etc., it is often inconvenient because you cannot view the recording due to the nature of real-time lectures.”
	Reduced concentration	15 (19)		Requires more effort to concentrate; Easier to get distracted		“It is easier to not concentrate or to sneak away and do other things during class.” “The duration of concentration does not last long.”
	Limited interaction	10 (13)		Limited interaction with classmates and lecturers, hard to speak up, and feeling disconnected		“It is difficult to actively communicate and interact with professors and students.” “It seems like student participation in class has dropped significantly.”

**Table 5:** Perceived advantages and disadvantages of asynchronous web-based learning (n=266).

Theme and perceived advantages and disadvantages		Values, n (%)	Description	Excerpts from written responses
**Advantages**				
	Unlimited access to lecture content	74 (27.8)	Repeated learning is possible and reviewing any particularly difficult part of the lecture is possible	“If there’s something you don’t know, you can rewatch it at any time. It was very helpful to understood some parts.” “It’s good because you can repeat the class and have time to pause and think when necessary.”
	The autonomy to choose one’s preferred learning time	69 (25.9)	Attending to classes when one is prepared and when one can focus the most and the option to progress through the class at one’s own pace	“Because I can study when I am ready, I have deeper concentration and understanding.” “I have the advantage of being able to pause and listen if I lack concentration.” “Learning online helps me learn at my own pace and in my own way, which helps boost my motivation.”
	Protective measures during the pandemic	48 (18)	Reduced risk of COVID-19, maintaining social distancing, not a face-to-face learning, and no need to wear facial masks	“Protecting the safety of students and instructors is of utmost importance in this time.” “The spread of coronavirus can be prevented in college-related populations.”
**Disadvantages**				
	Difficulties with self-regulated learning	66 (24.8)	Difficulty in attending classes consistently and one must manage their own learning well	“Too much freedom making me lazy.” “Pushing away from studying and anxiety right before exams.”
	Limited interaction	55 (20.7)	No real-time feedback, difficulty in asking questions, lack of direct communication, and feeling disconnected	“The downside is that it is difficult to immediately resolve questions when they arise. It can be only resolved through email or other communication methods.” “Lack of interaction between professors and students makes it hard to form a relationship.” “Unable to go to school or meet classmates.”
	Increased class assignments	27 (10.2)	Instructor assigns more assignments to students for learning management	“Since attendance could not be confirmed non-face-to-face, a lot of homework was given to prove attendance.” “Sometimes I felt burdened because there were too many assignments in one day.”

Regarding the advantages, both students in synchronous and asynchronous learning consistently identified heightened autonomy as a positive aspect of web-based learning. However, there were nuanced differences between the 2 settings. The students in synchronous settings emphasized autonomy in the “learning environment” (19/79, 24%), whereas those in asynchronous settings stressed the flexibility of “learning in preferred time” (69/266, 25.9%):

Taking classes in my own comfortable environment, optimized to learn the most, is the best thing of online learning.

Student #18, synchronous setting

Learning online helps me learn at my own pace and in my own way, which helps boost my motivation.

Student #23, asynchronous setting

Moreover, a shared perception among students in both synchronous (14/79, 18%) and asynchronous (48/266, 18%) settings was that web-based learning served as a protective measure to reduce the risk of infection for themselves and their families:

I feel safe because I don’t have to go to a crowded environment.

Student #56, synchronous setting

Protecting the safety of students and instructors is of utmost importance in this time.

Student #10, asynchronous setting

However, beyond these advantages, challenges in self-management of learning were also evident. The students in synchronous settings mentioned the effort required to concentrate during class (15/79, 19%). In contrast, the students in asynchronous settings highlighted the advantage of learning at their preferred times (69/266, 25.9%):

The duration of concentration does not last long.

Student #6, synchronous setting

Because I can study when I am ready, I have deeper concentration and understanding.

Student #110, asynchronous setting

Technical challenges were more pronounced in the synchronous setting (28/79, 35%), with issues such as malfunctioning microphones and speakers. In contrast, limited interaction emerged as a more common response among students in asynchronous settings (55/266, 20.7%), expressing difficulty in forming relationships because of a lack of interaction between professors and students:

There are times when the instructor’s microphone, speaker, etc. do not work properly.

Student #123, synchronous setting

Lack of interaction between professors and students makes it hard to form a relationship.

Student #203, asynchronous setting

## Discussion

### Principal Findings

The study’s findings revealed student satisfaction and experiences with web-based learning, particularly regarding TD, during the abrupt shift from traditional learning to web-based education in the early stages of the COVID-19 pandemic. Given the determinants of TD, higher self-regulated learning, learner-instructor interaction, and learner-content interaction were related to higher satisfaction levels. The learning structure (synchronous vs asynchronous) did not reveal a significant association with satisfaction. Nevertheless, the qualitative data analysis results of this study clearly illustrated the advantages and disadvantages of web-based classes in both synchronous and asynchronous structures. Notably, the high efficacy belief scores that web-based learning is an effective means of reducing the risk of COVID-19 were related to increased satisfaction levels with web-based learning.

Several findings from the results are worth noting. Learner-content interaction was the strongest predictor of student satisfaction, which is consistent with the results of previous studies that were conducted before the pandemic [29,37]. Learner-content interaction refers to a 1-way process of reflecting on the course content or course material, and it occurs when learners talk or think about the knowledge and information comprising the course experience [29,38]. Learner-content interaction is an essential cognitive process for organizing and reflecting on new knowledge by integrating prior knowledge [22,38]. Students spend most of their time on the required reading or assignments; thus, they should be supported and receive timely and sufficient learning materials and content.

Although numerous studies identified 2-way communication, such as “learner-learner” [39,40] and “learner-instructor” interactions [39,41-43], as the most important contributors to satisfaction with web-based learning, learner-learner interaction had a weak relationship with satisfaction in this study. A possible explanation for this finding may lie in the specific course objectives and instructional goal orientation. Learner-learner interaction is relevant to group discussions, group projects, and idea sharing, which occur during collaborative activities [40]. In addition, limited opportunities for interaction between students and instructors during web-based learning represented a significant barrier to web-based learning during the first year of the pandemic [44-46]. This study was conducted in the first semester of implementing web-based classes because of the COVID-19 pandemic; therefore, the limitation of interaction may also be applicable in this case.

Regarding learner-instructor interaction, the students’ desires or expectations regarding interaction with instructors may be low, leading to less impact on satisfaction with web-based learning. Students’ expectations are closely related to their values and preferences, which directly influence their perception of learning experiences. From the perspective of fulfillment theory, satisfaction is achieved by obtaining an agent’s desired state of affairs [47]. Even before the pandemic, students in South Korea typically refrained from being active in class by asking questions because of the psychological stress and cultural norms that inhibited active participation [48]. The educational environments in South Korea are not conducive to training students for questioning and discussion. In contrast, lectures with examination-oriented evaluations bring a competitive atmosphere to the class and only require students to listen to instructors. Therefore, the interactions between instructors and students are frequently low in South Korea [49], and there is a tendency of students to prefer web-based chats or calls rather than traditional forms of communication [50].

This study emphasizes the significance of efficacy beliefs, specifically individuals’ perceptions of the effectiveness of web-based learning in mitigating the risk of COVID-19, as a significant predictor of satisfaction. Additionally, one-fifth of the students surveyed indicated that the primary advantage of web-based education was its efficient and effective protection against COVID-19. Given that students perceived the likelihood of contracting COVID-19 to be on par with equal probability and perceived the severity of the virus to be high, efficacy beliefs likely played a crucial role in their willingness to adopt web-based learning. Numerous studies have tested the effect of efficacy beliefs on prevention behaviors for COVID-19 [1,25,26], cancer [51], driving [52], and the risk of using chemical products [53]. Therefore, although web-based classes were not the students’ preferred choice, firmer efficacy beliefs could lead to high levels of satisfaction with web-based learning. Similarly, previous studies have shown that psychological safety and perceived usefulness in web-based medical education are crucial to student satisfaction with web-based learning [54-56]. Therefore, this study’s results suggest that schools must try to identify a shared understanding and consensus with faculty, staff, students, and parents about the need for web-based learning to ensure their safety against public health emergencies such as COVID-19 to increase satisfaction. In other words, to manage the increasing anxiety among school members and to help reach an understanding among them, providing concrete grounds for decisions through open and sufficient communication is necessary for the school leadership [57].

The results of this study provide implications for education and future research. First, understanding the differences between synchronous and asynchronous learning when selecting a learning structure for web-based learning is essential. A distinction was noted between the perceptions of students regarding the advantages and disadvantages of web-based learning structures. Both students from synchronous and asynchronous learning cited autonomy of learning as an advantage of web-based learning. However, it is noteworthy that although students from synchronous learning predominantly mentioned the autonomy of location, students from asynchronous learning primarily highlighted the advantage of time management. In addition, synchronous teaching is preferred when peer interactions are required and critical thinking skills are taught at the novice level, whereas asynchronous instruction can facilitate autonomy among students [58,59]. Hybrid learning, which uses synchronous and asynchronous elements, may be effective for teaching integrated content or clinical scenarios [60]. Moreover, it may facilitate learner-instructor and learner-learner interactions and self-regulated learning [61]. Selecting between synchronous and asynchronous structures is critical for educators and students [20,62].

Second, communication models should be developed to facilitate 2-way interaction in web-based learning. Scholars have introduced several communication models for instructors and students in distance learning environments [63,64]. However, these models focus on the examination and content analysis of interactions instead of facilitating or educating instructors. Encouraging 2-way communication (eg, requiring students to keep their “cameras on” to foster appropriate videoconferencing behavior) [65] and using appropriate communication tools [66] can encourage students to contact instructors when they require further assistance. Moreover, efforts to strengthen students’ self-regulated learning are important. This study demonstrated that self-regulated learning influences affective outcomes, such as satisfaction and academic achievement or performance [29,67]. Web-based learning’s flexibility requires students to use additional self-regulatory skills [68], which can be taught before starting an web-based course or by providing support for skill development within the course itself.

This study has several limitations. First, we did not investigate other forms of interaction in web-based learning, which were suggested as additional factors to be incorporated into Moore’s model [19], such as learner-interface [69], learner-task [70], and learner-tool [71] interactions. Additional studies examining the influence of these interactions are required. Second, despite being conducted at >1 institution, the study could not investigate the potential predictors of satisfaction at the institutional level, such as tuition. Factors related to the institutional level may contribute to student satisfaction, which warrants further study.

Several studies have examined objective learning outcomes during the pandemic, such as grade point averages and test scores [8,72]. Subjective outcomes such as student satisfaction are equally important and of increasing interest [9-11]. Student satisfaction reflects the extent to which the learner positively perceives learning experiences and is an essential indicator of program-related and student-related outcomes, such as increased persistence and commitment to the program [73-75]. However, future studies investigating both subjective and objective learning outcomes could provide valuable implications for enhancing web-based learning.

### Conclusions

A call has been issued for dental educators to share their stories, practices, and experiences during the COVID-19 pandemic. The results of this study indicate that designing a learning structure to effectively achieve specific course objectives and facilitate student interactions is essential. Interactions between students and content were the most predictable factors for satisfaction. Therefore, support should be provided to ensure timely and sufficient learning materials and content. Furthermore, support is needed to strengthen the self-regulated learning of students. The results of this study can inform decision-making related to future policies, practices, and research. Moreover, understanding students’ perceptions of and satisfaction with web-based learning will aid policy makers and university authorities in planning and managing dental education to prepare for future crises.

## Abbreviations

DDS: Doctor of Dental Surgery
OCSS: Online Course Satisfaction Scale
TD: transactional distance

## Data Availability

The data sets generated during this study are not publicly available owing to the limitations of ethics approval involving student data and anonymity but are available from the corresponding author on reasonable request.
